# Going beyond personal protection against mosquito bites to eliminate malaria transmission: population suppression of malaria vectors that exploit both human and animal blood

**DOI:** 10.1136/bmjgh-2016-000198

**Published:** 2017-04-26

**Authors:** Gerry F Killeen, Samson S Kiware, Fredros O Okumu, Marianne E Sinka, Catherine L Moyes, N Claire Massey, Peter W Gething, John M Marshall, Carlos J Chaccour, Lucy S Tusting

**Affiliations:** 1 Environmental Health and Ecological Sciences Department, Ifakara Health Institute, Dar es Salaam, United Republic of Tanzania; 2 Department of Vector Biology, Liverpool School of Tropical Medicine, Liverpool, UK; 3 School of Public Health, University of the Witwatersrand, Johannesburg, South Africa; 4 Department of Zoology, University of Oxford, Oxford, UK; 5 Oxford Big Data Institute, Li Ka Shing Centre for Health Information and Discovery, University of Oxford, Oxford, UK; 6 Divisions of Biostatistics and Epidemiology, School of Public Health, University of California, Berkeley, California, USA; 7 Instituto de Salud Global, Barcelona Centre for International Health Research (CRESIB), Hospital Clínic, Universitat de Barcelona, Barcelona, Spain; 8 Instituto de Salud Tropical, Universidad de Navarra, Pamplona, Spain

## Abstract

Protecting individuals and households against mosquito bites with long-lasting insecticidal nets (LLINs) or indoor residual spraying (IRS) can suppress entire populations of unusually efficient malaria vector species that predominantly feed indoors on humans. Mosquitoes which usually feed on animals are less reliant on human blood, so they are far less vulnerable to population suppression effects of such human-targeted insecticidal measures. Fortunately, the dozens of mosquito species which primarily feed on animals are also relatively inefficient vectors of malaria, so personal protection against mosquito bites may be sufficient to eliminate transmission. However, a handful of mosquito species are particularly problematic vectors of residual malaria transmission, because they feed readily on both humans and animals. These unusual vectors feed often enough on humans to be potent malaria vectors, but also often enough on animals to evade population control with LLINs, IRS or any other insecticidal personal protection measure targeted only to humans. *Anopheles arabiensis* and *A. coluzzii* in Africa, *A. darlingi* in South America and *A. farauti* in Oceania, as well as *A. culicifacies* species E, *A. fluviatilis* species S, *A. lesteri* and *A. minimus* in Asia, all feed readily on either humans or animals and collectively mediate residual malaria transmission across most of the tropics. Eliminating malaria transmission by vectors exhibiting such dual host preferences will require aggressive mosquito population abatement, rather than just personal protection of humans. Population suppression of even these particularly troublesome vectors is achievable with a variety of existing vector control technologies that remain underdeveloped or underexploited.

**Key questions**

**What is already known about this topic?**

- The only significant infectious reservoirs for the two most common human malaria parasites are other humans, so most of the world's infection burden is mediated by a small number of highly efficient vector mosquitoes that predominantly feed on humans.
- Fortunately, these extraordinarily efficient malaria vectors are also highly vulnerable to attack with insecticidal personal protection measures for humans, such as long-lasting insecticidal nets (LLINs) and indoor residual spraying (IRS), which can suppress or even eliminate entire populations of such human-dependent mosquitoes.
- A larger number of malaria vector species strongly prefer feeding on animals, so they are far less vulnerable to population suppression with LLINs, IRS or any other insecticidal personal protection measure. However, they are also far less efficient vectors, so personal protection alone may be sufficient to eliminate the transmission they mediate.

**What are the new findings?**

- If malaria is ever to be eliminated, the one of the greatest vector control challenges ahead is presented by a small number of vector species which feed readily on both humans and animals. Mosquitoes with such flexible, dual feeding preferences can feed frequently enough on humans to mediate intense residual malaria transmission, but often enough on animals to evade mass population suppression with LLINs, IRS or any other insecticidal personal protection measures for humans.
- *Anopheles arabiensis* and *A. coluzzii* in Africa, *A. darlingi* in South America and *A. farauti* in Oceania, as well as *Anopheles culicifacies* species E, *A. fluviatilis* species S, *A. lesteri* and *A. minimus* in Asia, all feed readily on either humans or animals. Collectively, these eight species dominate residual malaria transmission across most of the tropics.

**Recommendations for policy**

- Eliminating malaria transmission by these eight exceptionally important vectors will require aggressive mosquito abatement, to kill entire vector populations en masse, rather than just personal or household protection of humans.
- A number of existing and emerging vector control technologies are available, which target mosquitoes when they feed on animals or during other life cycle stages, and could achieve population abatement of even these particularly troublesome vectors.
- Development and evaluation of these underexploited alternatives to LLINs and IRS is urgently needed, and requires immediate strategic investment if the long-term goal of malaria eradication is ever to be achieved.

## Introduction

The global distribution of malaria is overwhelmingly determined by environmental factors, particularly climate and the behavioural characteristics of local mosquito vectors.^1^ ^2^ The two most important species of human malaria parasites (*Plasmodium falciparum* and *P. vivax*) are both strict anthroponoses with no significant animal reservoir, so the more a mosquito species feeds on humans, the more efficient it will be as a vector of malaria.^1–3^ The vast bulk of the world's malaria burden therefore occurs in the poorest, least-developed countries of Africa and Oceania, where a small number of unusually efficient malaria vectors have evolved to specialise in feeding on humans.^1^ ^2^ Fortunately, this exceptional propensity to attack people also makes them vulnerable to attack with insecticidal measures for protecting humans against bites.^2^ ^4^

### Population suppression of human-dependent vectors through insecticidal personal protection

Malaria vector control with long-lasting insecticidal nets (LLINs) and/or indoor residual spraying (IRS) accounts for most of the malaria cases and malaria-related deaths averted over recent years.^5^ ^6^ These strategies can provide far more than just personal protection, by suppressing the densities and survival rates of entire vector populations.^7–9^ This mosquito population suppression function is often referred to as mosquito population *abatement* and causes an epidemiological *mass effect* on malaria transmission across entire communities. While the mosquito population abatement function of LLINs and IRS is less conceptually obvious than the benefits of personal protection, it probably accounts for most of the impacts^8^ seen in parts of Africa, Papua and Oceania where transmission has historically been extremely intense.^10^

**Figure 1:**
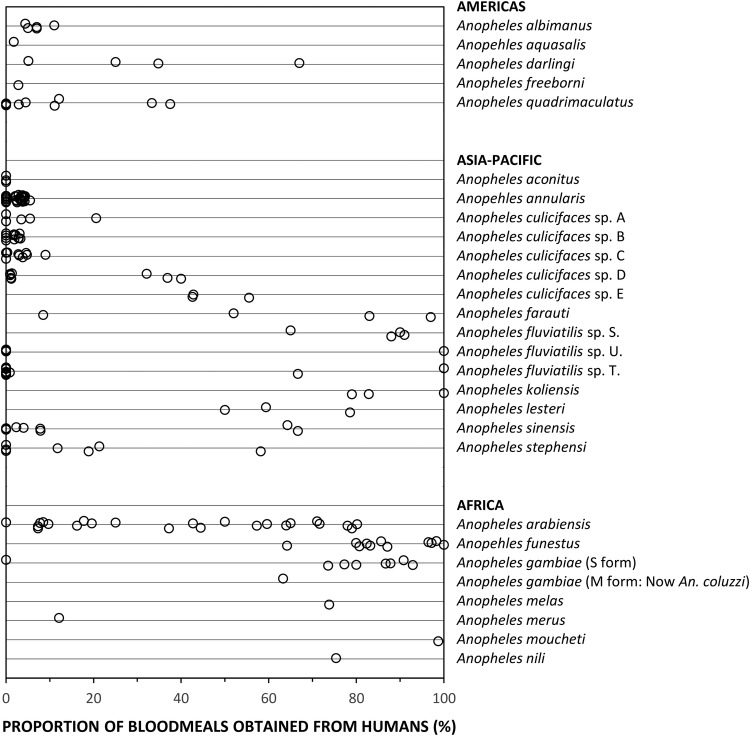
The proportions of blood meals obtained from humans by malaria vectors from the Americas, Asia, the Pacific and Africa. A recently published compilation of bionomic data for the world's most important vectors^62^ was filtered to exclude records representing undifferentiated mixtures of species from groups or complexes. In almost all cases, only records with estimates based on combined indoor and outdoor samples of mosquitoes were used. However, in the specific cases of *Anopheles farauti* and *A. culicifacies* species D, for which no data combining indoor and outdoor-caught samples were available, estimates based on outdoor-caught samples only were used. Also, for *A. farauti*, for which only one data point for sibling species-specific data was available from the contemporary data set, additional data was included from a historical study in which this species was identified morphologically in a setting where none of the other sibling species were present.^36^

LLINs and IRS have been so successful in these specific regions because they are home to a small number of highly-specialised vector species, exhibiting unusual behaviours that set them apart from the dozens of other *Anopheles* capable of mediating malaria transmission.^2^ ^4^ *Anopheles funestus* and *A. gambiae* in Africa are both extremely efficient malaria vectors and extremely vulnerable to attack with IRS and/or LLINs, because they are behaviourally adapted to exploit sleeping humans as their preferred blood source.^2^ ^4^ ^11^ Figure 1 illustrates how *A. funestus* and *A. gambiae* consistently feed predominantly on human blood throughout their range. This, together with the fact that these two vector species tend to bite humans while they are sleeping indoors in the middle of the night,^12^ means that IRS and LLIN campaigns can have dramatic effects on both these species.^2^ ^4^ ^11^ Indeed both species have been eliminated or almost eliminated from a range of African settings with LLINs or IRS.^2^ ^4^ In the Pacific, *Anopheles punctulatus*, as well as its sibling species *A. koliensis* and *A. farauti*, can readily feed on pigs, but such alternative hosts are scarce across much of their range, so they also often rely predominantly on humans for blood (figure 1). *An. farauti* can persist despite high coverage with LLINs and IRS, even where pigs are scarce, by feeding on humans outdoors in the early evening.^4^ ^13^ However, *A. punctulatus* and *A. koliensis* that feed at night when humans are indoors can be very vulnerable to these measures, and have even been eliminated from some of the Solomon Islands.^4^

It has long been noted that feeding in the middle of the night, when most people are usually asleep indoors, appears to be a behavioural specialisation of mosquitoes which regularly feed on humans.^2^ ^14^ Indeed it is the late-night foraging activity peaks of African vectors, rather than any particular preference for feeding indoors, that cause most of their encounters with humans to occur indoors so LLINs and IRS are highly effective.^12^ While these preferred nocturnal feeding times ensure that protection measures like LLINs and IRS effectively target most of the times and locations when exposure would otherwise occur, it is the associated strong preference for humans that ensures that protection for the human user reduces vector survival at population level.^2–4^ ^15–17^

**Figure 2:**
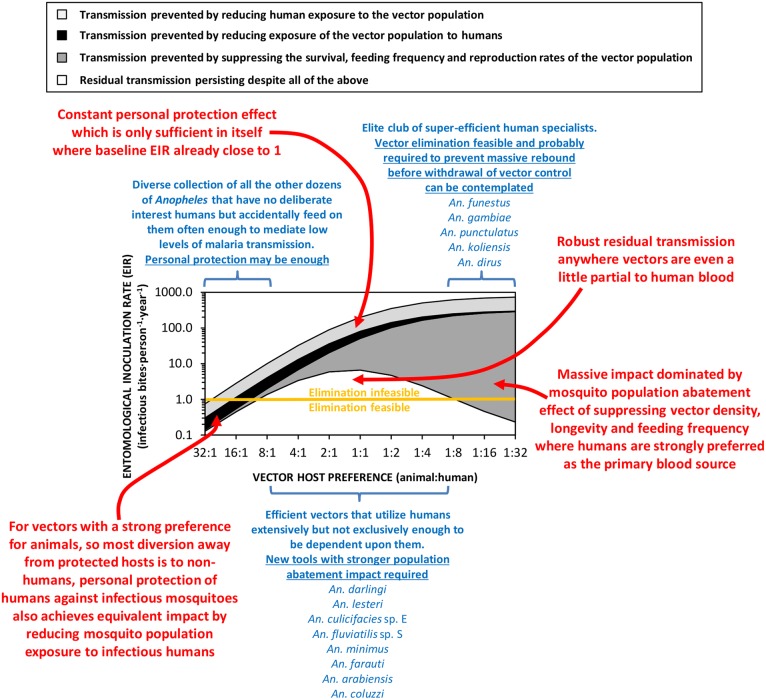
A schematic illustration of how malaria transmission intensity and responsiveness to personal protection varies according to vector preference for animals rather than humans.^2–4^ ^15–17^ The simulations were implemented as previously described,^2^ ^3^ except that the overall impact of personal protection measures (equivalent deterrent and insecticidal properties to a typical modern long-lasting insecticidal net assumed) are presented broken down by contributing underlying mechanism, and an Allee effect was incorporated.^4^ The entomological inoculation rate threshold below which elimination of malaria transmission may be feasible with existing diagnostic and therapeutic technologies (Orange horizontal line), was defined based on the most recent authoritative modelling studies.^31^

In fact, the vulnerability to IRS and LLINs of *A. funestus* and *A. gambiae* in Africa, as well as *An. punctulatus* and *A. koliensis* in the Pacific, is so striking that it has been suggested that Allee effects may occur in mosquito populations.^4^ Allee effects are widespread among a diversity of animals, plants and microbes, and arise when the fitness of individuals depends on overall population size or density.^18^ ^19^ Consequently, a population can rapidly collapse once pushed below a certain minimum density, without entirely comprehensive further persecution.^18^ ^19^ So while these highly efficient vectors that predominantly feed on humans remain public health enemy number one, they can be effectively controlled and ideally eliminated,^4^ through massive population abatement effects of LLINs (figure 2), IRS, or emerging technologies designed to supersede them.^20^ It should also be possible to achieve population suppression or elimination of the similarly human-specialised and efficient vectors that bite outdoors, such as exophagic populations of *Anopheles dirus* in south east Asia,^21^ with lethal insecticides deployed as clothing treatments or vapour emanators.^20^

### Residual malaria transmission by mosquitoes which feed on animals

The obvious Achilles heel of this public health miracle is that the mosquito population abatement functions of both LLINs and IRS rely on unusually strong vector preferences for feeding on sleeping humans and/or resting inside human habitations.^2–4^ ^15–17^ An inevitable corollary of this principle is that LLINs and IRS are therefore poorly-suited to tackling the much larger number of important vector species that usually feed on animals (figure 2). Many such *zoophagic* species also feed and/or rest outdoors, and/or forage only briefly and cautiously within houses when they do enter them.^14^ ^22–26^ So not only does the intensity of malaria transmission vary according to the preference of local mosquito populations for human versus animal blood, so does the responsiveness of transmission to insecticidal interventions which protect people against bites. Here we synthesise the literature and exploit existing process-explicit models to examine how feeding on animals, rather than humans, influences the level of impact on malaria transmission that is needed to eliminate it, and can be feasibly achieved by directly protecting humans against mosquito bites.

### The challenges of controlling malaria vector mosquitoes that feed on animals

In high-intensity transmission systems which historically had multiple vectors, effective suppression of species like *A. gambiae* and *A. funestus* in Africa or *A. punctulatus* and *A. koliensis* in the Pacific, has left less vulnerable species like *A. arabiensis* or *A. farauti*, respectively, to dominate and sustain *residual* malaria transmission (box 1). The most important trait that all these mosquitoes appear to have in common is the ability to feed flexibly on either humans or animals, depending on availability of these alternative blood sources.^13^ ^27^ ^28^ Also, it has long been known that behavioural tendencies of mosquitoes to feed outdoors at dusk and dawn, and then rest outdoors afterwards, are usually associated with preference for feeding on animals.^2^ ^14^ Furthermore, the short feeding and resting times that can allow mosquitoes to feed on insecticide-treated cattle,^29^ or to enter but then safely escape from houses containing LLINs and/or IRS,^14^ ^22–26^ also appear to occur predominantly among zoophagic mosquitoes,^2^ ^14^ ^30^ possibly because animals exhibit more active defence behaviours than sleeping humans.^30^ Mosquitoes that can be described as at least partially zoophagic are therefore particularly important vectors of residual malaria transmission, because they can readily feed on animals wherever they are available in sufficient numbers, and often exhibit outdoor feeding, outdoor-resting and early-exit behaviours that also limit their vulnerability to LLINs and IRS. That said, they nevertheless feed on humans with sufficient frequency to maintain stable malaria transmission (figure 2).

Box 1 **Residual malaria transmission**

Residual malaria transmission is defined by the WHO as “Persistence of transmission after good coverage has been achieved with high-quality vector control interventions to which local vectors are fully susceptible”.^57^ However, here we define it more specifically for the purposes of this analysis.

**Definition:**^2^ Residual malaria transmission is any component of ongoing transmission that can persist after scaling up long-lasting insecticidal nets (LLINs) and indoor residual spraying (IRS), with active ingredients to which local vectors are fully physiologically susceptible, to universal coverage targets.^8^ ^9^

**Implications:** This more explicit definition of residual transmission therefore represents a fundamental, purely biological *limitation* to the level of impact that can be reasonably expected of LLINs or IRS, caused by specific behaviours of mosquitoes and humans. Put simply, no insecticidal technology can protect people who do not use it when they are exposed to mosquitoes, or kill mosquitoes that avoid physical contact with its active ingredients.

Residual malaria transmission is therefore distinct from other important causes of ongoing malaria transmission, including financial or operational failures to achieve sufficiently high coverage with LLINs/IRS,^10^ ^45^ ^58^ ^59^ or lack of insecticidal active ingredients to which the vector remains fully physiologically susceptible.^60^ ^61^

### Opportunities for personal protection measures to eliminate malaria transmission by vectors which strongly prefer animal blood

Since *P. falciparum* and *P. vivax* are both strict anthroponoses, with no significant zoonotic animal reservoir, strongly zoophagic mosquitoes with strong preferences for animal blood are far less efficient vectors than those preferring human blood (figure 2). Most of the world's known malaria vectors match this profile (figure 1) and appear to only bite humans occasionally.^2^ Such infrequent feeding on humans can nevertheless be sufficient to mediate self-sustaining transmission intensities, in excess of one inoculation per person per year (figure 2). Population suppression of highly zoophagic mosquitoes cannot be reasonably expected from personal protection measures like LLINs and IRS deployed indoors, or from insecticide-treated clothes and vapour-phase insecticides deployed outdoors, because humans are a negligible fraction of the blood resources that sustain them.^3^ ^15–17^

However, such strong preferences for feeding on animals do enable a mass effect through reduced human-vector contact, regardless of whether the protective measure actually kills mosquitoes or merely deters them away from the user to seek blood elsewhere:^3^ ^16^ ^17^ When zoophagic mosquitoes are frustrated but not killed while attempting to feed on protected humans, on most occasions they will consequently feed on an animal rather than an unprotected human. Not only are human populations protected against infectious mosquitoes, mosquito populations are also protected against exposure to infectious humans.^3^ ^16^ ^17^ Also, strong zoophagy inevitably results in only modest vectorial capacity, so mass suppression of entire vector populations may not be necessary: It may well be possible to effectively tackle the low levels of transmission mediated by these vectors (figure 2) by supplementing existing diagnostic and therapeutic technology^31^ with emerging new technologies for personal protection of humans. Insecticide-treated clothes and long-lasting emanators for vapour-phase insecticides or repellents that can be deployed when people are active outdoors may be especially useful.^20^

### Resilient, adaptable, efficient vectors that exploit both human and animal blood

**Figure 3:**
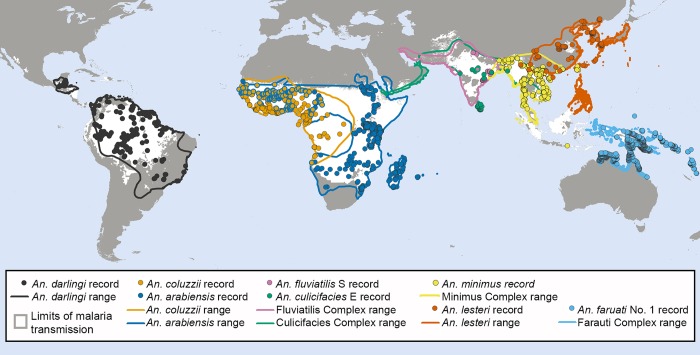
The global distribution of malaria vector species known to feed readily on either humans or animals. Records of mosquito occurrence identified to the sibling species level using molecular methods were extracted from the Malaria Atlas Project database.^62^ ^63^ The ranges for *Anopheles darlingi*, the *A. fluviatilis* complex, the *A. culicifacies* complex, *A. lesteri* and *A. farauti* complex were outlined using published data and expert opinions as previously described,^64^ ^65^ while that for the *Anopheles minimus* complex was adjusted to incorporate newer records. The range for *Anopheles arabiensis*, previously defined using expert opinions,^66^ was updated to encompass newer records of this species.^62^ To generate an approximate range for *Anopheles coluzzii* (formerly *A. gambiae* M Form), the previous range for *A. gambiae* and *A. coluzzii* combined was adjusted to capture the areas where *A. coluzzii* has been recorded and exclude those where only nominate *A. gambiae* (formerly *A. gambiae* S Form) has been reported, using both data from the Malaria Atlas Project and an earlier map of the M and S forms of *A. gambiae*.^67^ The resulting data and ranges were overlaid on a map showing the limits of *Plasmodium falciparum*^68^ and *P. vivax*^69^ transmission.

A key challenge in the field of malaria vector control in the years ahead will be to effectively tackle transmission by a small subset of *Anopheles* mosquitoes that do not rely heavily on either human or animal blood, but are instead capable of opportunistically exploiting either source of nutrition depending on availability (figure 2). *A. arabiensis* is probably the most important vector of residual malaria transmission in many parts of eastern and southern Africa, and has similarly strong preferences for both humans and cattle.^28^ The proportion of blood meals it obtains from humans is therefore predictably dependent on fine-scale variations in availability of these two host species,^28^ varies across a very wide range (figure 1), and can be dramatically reduced by LLINs if cattle are available as alternative hosts.^32^ In central and western Africa, the only reported estimate for the proportion of blood meal obtained from humans by *An coluzzii* is clearly below the range of reported values for its sibling species *A. gambiae* (figure 1). *A. coluzzii* can persist following LLIN/IRS scale up and dominate residual populations of the species complex,^33^ by switching to feeding on animals.^34^ Indeed, even *A. gambiae* itself is now resorting to obtaining blood from animals in parts of west Kenya where LLIN coverage has been high for some time.^35^ Only a single data point is available for each of the four more focally-distributed coastal (*Anopheles melas* and *A. merus*) and riverine (*A. moucheti* and *A. nili*) African species (figure 1). Nevertheless, except for *A. moucheti*, these limited observations confirm mixed feeding on humans and animals. In the Pacific, *A. farauti* survives despite deployment of LLINs and IRS, by combining biting in the early evenings with a ready ability to feed on pigs wherever they are available,^13^ ^36^ ^37^ thus exhibiting a similarly wide range of variation in its reliance on human blood to *A. arabiensis* (figure 1). *Anopheles darlingi* in Latin America often feeds on humans, but is far from reliant on humans as a sole blood source, so the proportion of blood meals it obtains from humans also varies considerably (figure 1). Notably, *A. darlingi* was one of the first vector species in which early-exiting behaviour was identified as a cause of residual transmission.^14^ Figure 1 also reveals *A. lesteri*, *Anopheles culicifacies* species E and *A. fluviatilis* species S as additional species that often feed frequently on humans, but do not depend exclusively on humans for their survival. This relatively high level of anthropophagy sets both *A. culicifacies* species E and *A. fluviatilis* species S apart from their sibling species in south-central Asia in terms of vectorial efficiency.^38^ Non-obligate ability to feed on humans also accounts for the dominance of *A. lesteri* over *A. sinensis* as a historical cause of malaria transmission in China.^39^ The ability of such vectors to exploit human blood wherever they can find it makes them far more efficient vectors than the more devoutly zoophagic vectors that constitute the majority of species in figure 1. However, following scale up of LLINs and/or IRS, their ability to exploit animal blood can allow them to survive and mediate resilient malaria transmission at far greater intensity than more efficient, previously-dominant, human-dependent vectors (figure 2), which may even become locally extinct.^4^ Collectively these species encompass most of the malaria-endemic world, so the issues raised here affect every region (figure 3).

Figures 1 and 3 reveal considerable limitations in available field data and even in the methodology available to address those limitations. Note that the data gaps apparent on the map in figure 3 reflect a lack of data for feeding preferences of the individual species, rather than definitive evidence for an absence of vector species that readily switch between humans and animals. For example, *A. punctulatus* is absent from figure 1 because all blood meal origin data reported thus far relate to the *A. punctulatus* complex. While direct experimental measurements of host preference^40^ provided evidence that led us to include *A. dirus* and *A. minimus* in figure 2, both species are absent from figure 1, presumably because blood-fed specimens of these outdoor-resting mosquitoes are notoriously difficult to capture. Correspondingly, the only contemporary data point for *A. farauti* in figure 1 arose from recent innovations in methods for sampling outdoor-resting mosquitoes.^41^

Despite these data limitations, the implication of widespread preferences for feeding on animals by malaria vector mosquitoes is clear. Not only does zoophagy allow mosquitoes to avoid humans in the first place, it is often associated with additional behavioural idiosyncrasies that enable them to avoid fatal exposure to LLINs and IRS whenever they do encounter humans.^2^ ^14^ ^25^ The simulations in figure 2 assume that, apart from host preference, all other behavioural traits, insecticide susceptibilities and effects of personal protection measures, are equivalent.^3^ However, two other behaviours of zoophagic mosquitoes can cause additional problems that are not accounted for in these simulations:^2^ ^42^ ^43^ (1) Feeding outdoors in the early evenings or mornings when people are active outside of their nets or houses, or (2) cautious, brief, but repetitive, foraging within houses until an exposed victim is encountered, whereby the vector exits too quickly from any individual house to be killed by IRS or LLINs.^14^ ^22–26^ This is particularly true of *A. arabiensis*, which may be considered a stereotypical vector of residual malaria transmission, because it exhibits all three of these behaviours.^26^ In some parts of South and Central America, the same appears to be true of *A. darlingi*^14^ ^44^ *A. farauti* is notorious for attacking people outdoors, often at times of the evening and morning when people are active so protection with IRS, LLINs or even insecticide-treated hammocks is impractical.^37^

This subset of adaptable and evasive mosquito species, which exploit both humans and animals with comparable relish, therefore represents a major vector control challenge to be overcome (figure 2) if we are ever to live in a malaria-free world.^45^ ^46^ Packages of interventions that can eliminate intense transmission by these remarkably resilient vectors will probably be more than sufficient to deal with both the weaker animal-specialised vectors on the left side of figure 2 and the efficient but vulnerable human specialists on the right. Improved methods for protecting humans outdoors, with insecticide-treated clothing or vapour-phase insecticides^20^ will probably be required to eliminate transmission by vectors belonging to the left hand side of figure 2 or the middle,^2^ ^16^ and there are even concerns about some to the right.^47^ ^48^ Conversely, improved methods of indoor control to supplement, improve on and ultimately supersede LLINs and IRS,^20^ will be required to prevent indoor exposure and achieve mass population abatement of vectors on the right hand side of figure 2 and those in the middle.

However, even personal protection packages for humans that cover them while indoors and outdoors may often be insufficient to eliminate transmission by vectors which are anthropophagic enough to mediate intense transmission but zoophagic enough to escape from the full impact of population abatement (figure 2). Insecticide-treated clothing and vapour emanators are the most conceptually obvious way to extend the population abatement impacts of existing LLIN and IRS interventions beyond indoor-feeding vectors.^20^ However, even these additional personal protection measures may be insufficient for tackling transmission by vectors listed in the middle of figure 2, which are both anthropophagic and zoophagic. Examples of elimination of transmission by species like *A. arabiensis*^49^ ^50^ and *A. darlingi*,^51^ have been documented at the edge of their ranges, but not under the kind of lowland, equatorial climatic conditions that occur at the centres of their ranges^52^ and were assumed for the simulations in figure 2.

### Conclusions

It therefore seems likely that eradication of malaria globally, including regions with vectors that are both zoophagic and anthropophagic (figure 3), will require more aggressive mosquito control measures, which go beyond personal protection of humans against bites to achieve mass population suppression. The most conceptually obvious way to extend the lethal effects of LLINs and IRS beyond humans, to kill even these troublesome zoophagic mosquito species, is to target them with existing veterinary insecticide products when they feed on livestock.^20^ Also, new methods are now emerging which target mosquitoes when they feed on sugar or aggregate into mating swarms, regardless of their blood-feeding behaviours.^20^ Indeed a promising array of new vector control products and prototypes, such as attractive sugar baits, vapour-phase insecticide emanators, veterinary insecticides and house entry traps, are now emerging that could be horizontally delivered to end users almost anywhere in some of the poorest countries in the world.^20^ Furthermore, all the adult mosquito behaviours which these intervention options target can be readily quantified with existing, accessible, well-established entomological field techniques.^53^ Such metrics of targetable mosquito behaviours may therefore be used to rationally select, monitor and evaluate optimal intervention choices, to maximise impact on malaria transmission.^53^

However, there are also immediate, substantive opportunities to develop and evaluate delivery systems in low income and middle income countries (LMICs) for well-established mosquito abatement technologies that are already deployed extensively in high-income countries (HICs). Larval source management and space spraying have been implemented in many HICs for decades,^54^ ^55^ so an impressive arsenal of off-the-shelf commercial products is readily available through a thriving market.^20^ These more aggressive, vertically-delivered vector control methods have also proven successful in several selected LMIC settings, but remain under-exploited generally.^20^ Programmatic implementation research is therefore urgently required to develop the kind of proactive, area-wide mosquito abatement programmes that many of us in HICs have come to take for granted as routine local government services.^54^ ^55^ Rather than ask *whether* these proven mosquito control interventions can work against malaria vectors, greater emphasis should be given to the questions of *where* and *how* to deliver these services effectively and sustainably in LMICs.^56^
